# Low-cost locally manufacturable unilateral imperial external fixator for low- and middle-income countries

**DOI:** 10.3389/fmedt.2022.1004976

**Published:** 2022-11-28

**Authors:** Mehdi Saeidi, Spencer C. Barnes, Michael A. Berthaume, Sander R. Holthof, Giovanni S. Milandri, Anthony M. J. Bull, Jonathan Jeffers

**Affiliations:** ^1^ Department of Bioengineering, Imperial College London, London, United Kingdom; ^2^ Department of Mechanical Engineering, Imperial College London, London, United Kingdom

**Keywords:** external fixator, LMIC, long bone, low-cost, medical device, open fracture, temporary fracture fixation

## Abstract

Treating open fractures in long bones can be challenging and if not performed properly can lead to poor outcomes such as mal/non-union, deformity, and amputation. One of the most common methods of treating these fracture types is temporary external fixation followed by definitive fixation. The shortage of high-quality affordable external fixators is a long-recognised need, particularly in Low- and Middle-Income Countries (LMICs). This research aimed to develop a low-cost device that can be manufactured locally to international standards. This can provide surge capacity for conflict zones or in response to unpredictable incidents and situations. The fixator presented here and developed by us, the Imperial external fixator, was tested on femur and tibia specimens under 100 cycles of 100 N compression-tension and the results were compared with those of the Stryker Hoffmann 3 frame. The Imperial device was stiffer than the Stryker Hoffmann 3 with a lower median interfragmentary motion (of 0.94 vs. 1.48 mm). The low-cost, easy to use, relatively lightweight, and easy to manufacture (since minimum skillset and basic workshop equipment and materials are needed) device can address a critical shortage and need in LMICs particularly in conflict-affected regions with unpredictable demand and supply. The device is currently being piloted in three countries for road traffic accidents, gunshot wounds and other conflict trauma—including blast cohorts.

## Introduction

Every year, there are millions of open long bone fracture cases in Low- and Middle-Income Countries (LMICs) due to high-energy trauma, e.g., traffic accidents and conflict injuries (1–4). Approximately 70% of fractures in LMICs are caused by traffic accidents (5), yet this hides the local effects in warfare where fractures may be exclusively caused by conflict. As an example, the Head of plastic and reconstructive surgery in Gaza reported more than 300 high-energy compound tibial fractures in June 2018 alone (6). In India, approximately 4.5 million open fractures occur annually (7).

Given the severity of the soft tissue damage as well as the lack of advanced resources and healthcare training, external fixators play a critical role in treating these fractures in LMICs (8, 9). However, as well as the lack of affordability of most commercial fixators in these countries, there is also a lack of availability (10, 11), resulting in the creation of homemade external fixators in conflict zones (12). One approach to addressing this deficit is through donations (13). However, there is evidence from multiple conflict zones, including Ukraine (14), that cost (15) and immediate surge capacity at the start of conflicts and in conflicts where supply chains are disrupted result in significant shortfall (16).

To address the critical needs raised above, the aim of this study was to develop a low-cost, locally manufacturable unilateral external fixator (the Imperial external fixator) for LMICs. Not only should this device address the general shortage in those countries, but also enable the surge capacity which is particularly imperative in conflict regions or unforeseeable events, such as the sudden escalation (May 2021) in the Gaza war with nearly 2,000 casualties and no possibilities of importing humanitarian supports (17), the Beirut explosion (August 2020) with over 5,000 injured and a significant shortage of medical supplies (18), and the war in Ukraine (2022).

## Materials and methods

### Specifications

This research was conceived due to a shortage of affordable external fixators in Sri Lanka reported by one of the partners, an orthopaedic surgeon. Following detailed fieldwork and documenting clinical and functional requirements, the following specifications were derived:

• relatively lightweight;
• easy to use/reuse;
• using readily-available material;
• manufacturable using conventional workshop equipment; and
• provide stiffness similar to commercial fixators.

As bone pins were found to be available and easily accessible, these were not included in the specification. The final specifications were communicated to colleagues in other partner locations, including Gaza, and these were confirmed as appropriate, thus permitting these to be considered general to LMICs.

### Design

During the project, several versions of the device were developed and modified according to the feedback received from the local partners and surgeons. The final design only is discussed in this paper and other designs were investigated by our partners in Sri Lanka (19).

**Figure 1:**
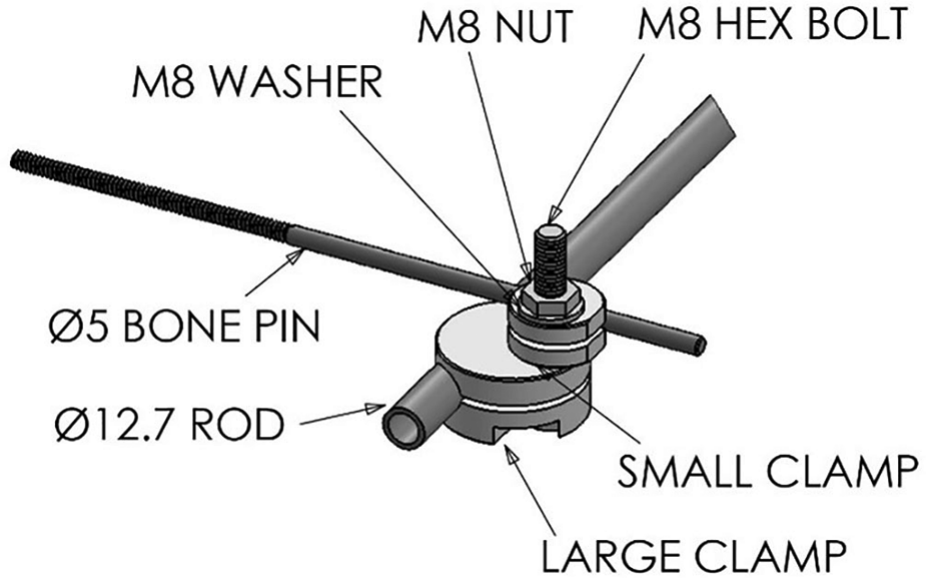
Components of a single clamp unit of the imperial external fixator.

In the standard configuration, each device used to fix one fractured long bone was comprised of four clamping systems and a rod, the component parts which are shown in Figure 1 and listed in Table 1. Technical drawings and further specifications for each part can be found on the official web page for this device: imperial.ac.uk/external-fixator/drawings.

**Table 1:** Imperial external fixator single clamp unit components.

Item	Qty
Aluminium 6xxx small slit clamp	4
Aluminium 6xxx large slit clamp	4
Stainless steel 304 tube	1
Stainless steel hex M8 × 40 mm bolt	4
Stainless steel hex M8 nut	4
Stainless steel M8 washer	4
5 mm Self tapping bone pin (off the shelf)	4

### Manufacturing

**Figure 2:**
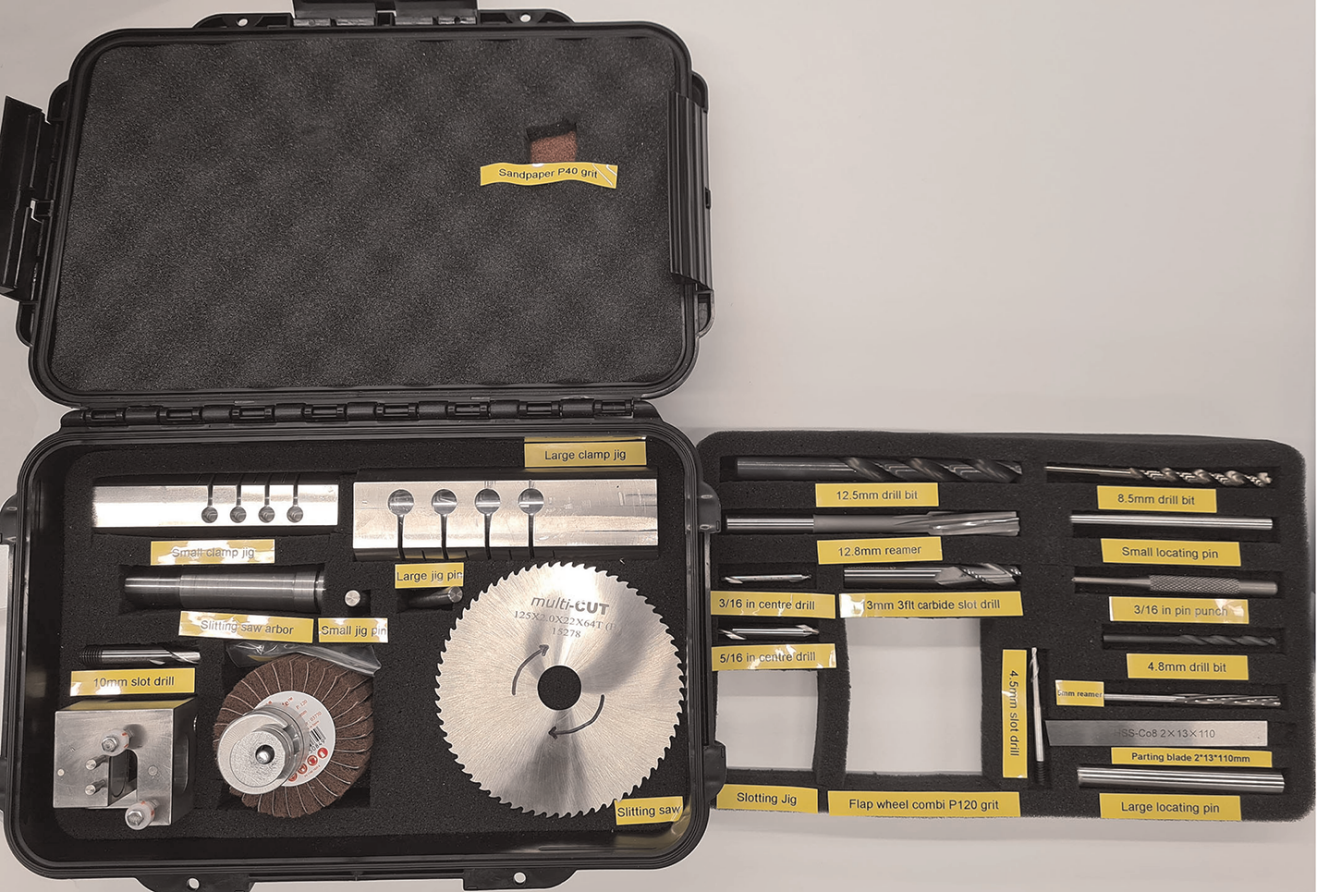
Imperial fixator manufacturing toolkit.

The device was designed to enable local manufacture in LMICs using conventional manufacturing techniques, i.e., turning and milling. However, during the testing process, it was identified that highly skilled operators were required, resulting in either high labour cost, or low-quality parts. Therefore, a manufacturing toolkit comprising three custom jigs and key required tool parts was also developed (Figure 2). The detailed specifications of the toolkit are available at: imperial.ac.uk/external-fixator/manufacturing.

In addition to minimising the required experience/training to produce the devices, using this toolkit leads to increased manufacturing accuracy, decreased manufacturing time, and a more standardised process, as well as being less dependent on the quality of the equipment. Mechanical tolerances are critical to the functioning of the device. In this study, all clamps were quality checked that they complied with the manufacturing tolerances given in the drawing.

### Cadaver testing

Cadaver testing was conducted on eight specimens, four femurs and four tibias from four donors (Table 2). The specimens were obtained from ScienceCare Anatomical Inc. (Phoenix, AZ, USA) with institutional ethics approval.

**Table 2:** Specimen donor details.

Age (years)	Height (m)	Body mass (kg)	Sex
63	1.67	70	F
62	1.62	60	F
58	1.60	60	M
62	1.65	53	M

Reflective markers (BrainLab, UK) were attached to the proximal and distal half of the bone using bi-cortical pins, and a Polaris optical tracking system (Vega, Northern Digital Inc, Canada) was used to track the movement of the bone segments throughout the experiment.

#### Femoral protocol

**Figure 3:**
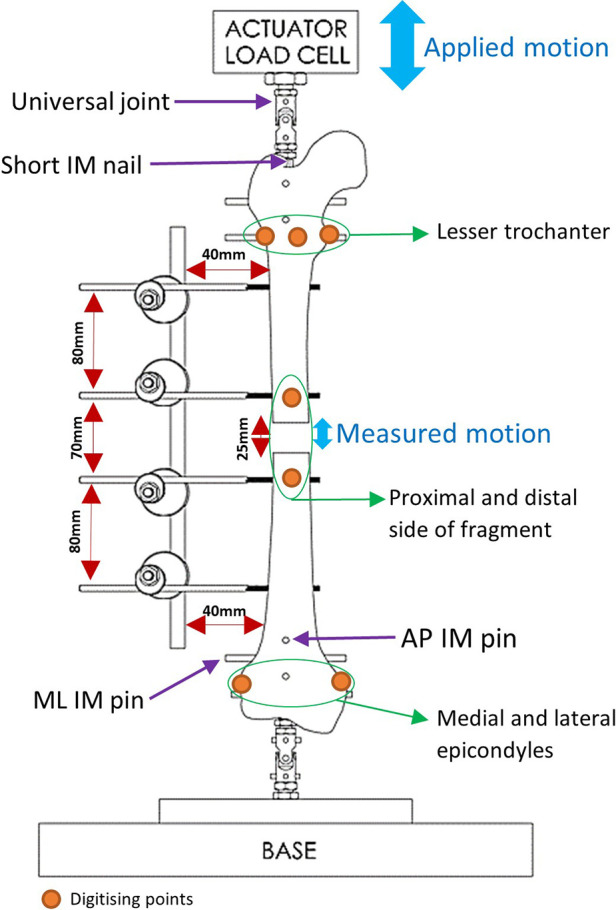
A schematic of the testing setup.

Fiducial marker screws were placed on the medial, anterolateral and posterolateral sides of the lesser trochanter, as well as the medial and lateral femoral epicondyles and the proximal and distal side of the fracture (Figure 3). These marker screws were then digitised and used to construct two axis systems, for the proximal and distal ends of the femur. The axes systems used the markers on the proximal and distal sides of the fragment as origins. The direction of the Proximal-Distal (PD) axis was defined as the line between the centre of the circle going through the markers on the lesser trochanter and the midpoint of the femoral epicondyles. The direction of the Mediolateral (ML) axis was defined as the line between the femoral epicondyles. The Anterior-Posterior (AP) axis was defined as the cross-product of the PD and ML axes. Using a custom MATLAB (MathWorks, USA) script, the movement of the proximal and distal ends of the fracture was calculated in these axis systems. Similar protocols have been used in studies to obtain 6 Degrees of Freedom (DOF) kinematics of knee joints (20–22). The tracking system has a translational accuracy of ±0.1 mm (23). The first 11 loading cycles were excluded to remove noise and build-up to cyclical movement.

Using the MATLAB script, the PD movement between the proximal and distal ends of the fracture was calculated by projecting the distance between the proximal and distal origin points onto the PD axis. The amplitude of the movement was then found by averaging the peak-to-peak PD movement across the loading cycles.

#### Tibial protocol

The tibial experiments followed the same protocol as the femur but used different landmarks to construct the axis systems. Markers were placed on the most medial and lateral points of the tibial plateau, and the medial and lateral malleolus of tibia and fibula (that was attached distally to the tibia, using a tricortical screw), and on the proximal and distal ends of the fracture. The direction of the PD axis was defined as the line between the midpoint of the medial and lateral points of the tibial plateau and the midpoint of the malleolar axis. ML axis direction was defined as the line between the medial and lateral points of the tibial plateau. The AP axis was the cross-product between the PD and ML axes. The movement between the proximal and distal ends was again projected onto the PD axis to find the amplitude of the PD movement.

### Mechanical testing

Testing for stiffness was performed according to ASTM F1541-17, Standard Specification and Test Methods for External Skeletal Fixation Devices. Specimens were tested under 100 cycles of 100 N compression-tension using an Instron machine and the interfragmentary motion was measured. Stryker Hoffmann®3 (Newbury, UK) was used as the benchmark during the testing process. Four Imperial and Hoffmann®3 fixators were used. The Imperial and Stryker devices weigh 450 and 370 g, respectively. Proximal and distal heads of each specimen were fixed using a short Intramedullary (IM) nail and four pins: two Anterior-Posteriorly and two Medio-Laterally. The specimen was then mounted on the Instron using universal joints (Figure 3).

**Figure 4:**
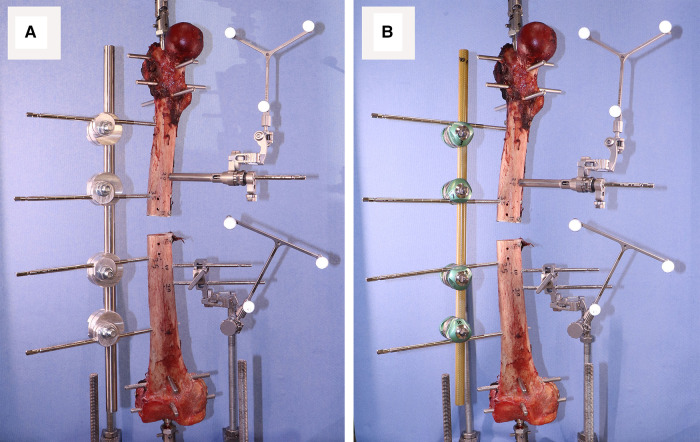
Mechanical testing of a femur with (**A**) imperial, and (**B**) Hoffmann 3 external fixators.

After inserting the bone pins into an intact specimen, the bone was mounted on the Instron and after installing the first fixator, a short segment of bone was cut out using an oscillating saw (Figure 4).

**Figure 5:**
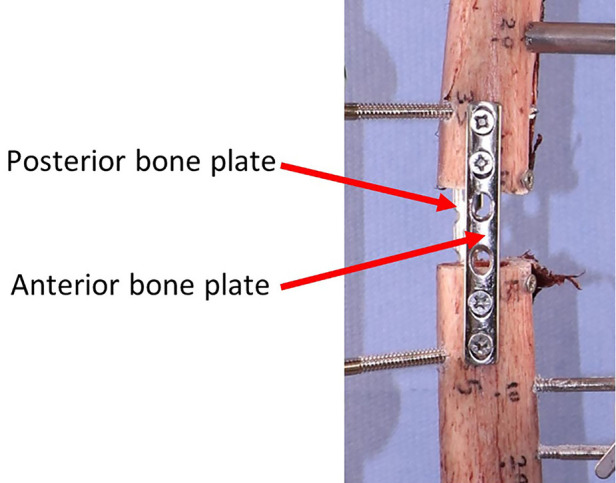
Securing the interfragmentary gap before removing the first device to install the second one.

Before removing the first device and installing the second one, the gap between proximal and distal parts of the bone was secured using two bone plates (positioned anteriorly and posteriorly) as shown in Figure 5.

### Statistical analyses

A Bayesian mixed-effects linear model was run to test for differences in interfragmentary motion between the two devices following the protocol set forth in McElreath (24). The “rethinking” package version 2.13 in R was used with RStudio (RStudio, PBC, Boston, MA) for statistical analyses (24, 25). A mixed-effects model was chosen to account for effects of: repeated measures on the same bone, using the femur or tibia of the same individual, which fixator was tested first, and that the fixators may become progressively looser with increasing number of cycles. The average interfragmentary motion was estimated using the following equation:

$$Avg.\;Interfragmentary\;motion=a+a_{bone}+a_{ind}+a_{test}+b_{cycle}*cycles+b_{device}*device$$

Where *a* is the intercept, *a_bone_* is the effect of bone (femur vs. tibia), *a_ind_* is the effect of individual (1–4), and *a_test_* is the effect of test (i.e., whether the device was tested first or second). Variable *b* is a slope, and *b_cycle_* quantifies changes in interfragmentary motion over the course of the test (e.g., due to loosening of the external fixator), and *b_device_* quantifies the effect of device (Imperial vs. Stryker). Only the last 59 cycles were used, as the first 31 cycles occasionally applied maximum tensile loads <96 N, and minimum compressive loads >−94 N.

Markov Chain Monte Carlo (MCMC) sampling (4 chains, 10,000 iterations, warmup = 2,000 iterations) with weakly informative priors were used, yielding posterior distributions with 8,000 iterations per chain (32,000 iterations total). When examining the posterior distributions, some parameters appeared insignificant (*p* > 0.05). Six additional statistical models were run leaving out parameters that appeared insignificant. Watanabe-Akaike Information Criteria (WAIC) was used to compare model performance and choose the best statistical model.

## Results

Measured peak-to-peak interfragmentary motions under cyclical loading are reported in Table 3. The Imperial fixator had a smaller interfragmentary motion than the Stryker fixator. This was due to the different material of the rod—the Imperial device was a stainless-steel tube whereas the Stryker device was a carbon composite rod.

**Table 3:** Measured mean interfragmentary motion in cyclical loading.

Specimen	L/R	Test 1		Test 2	
		Device	Interfragmentary motion (mm)	Device	Interfragmentary motion (mm)
Femur-S1	L	Stryker	0.97	Imperial	0.84
Femur-S2	R	Imperial	0.72	Stryker	1.35
Femur-S3	R	Imperial	1.33	Stryker	2.91
Femur-S4	L	Stryker	2.13	Imperial	1.23
Tibia-S1	L	Imperial	1.24	Stryker	1.67
Tibia-S2	R	Stryker	1.68	Imperial	1.32
Tibia-S3	R	Stryker	1.98	Imperial	1.24
Tibia-S4	L	Imperial	1.56	Stryker	2.23

To confirm the difference in interfragmentary motion was due to the fixator design and not another effect, the Bayesian model indicated that when the average effects of bone, individual, and test were used, the Stryker fixator has a median interfragmentary motion of 1.48 mm (95% confidence interval, CI: −0.60 to 4.11 mm) and the Imperial fixator has a median interfragmentary motion of 0.94 mm (95% CI: −1.15 to 3.57 mm). These statistical differences are derived from the WAIC criteria showing that the best model included all parameters except cycle (Table 4) and the effect of bone, individual, and test were non-significant as indicated by 95% CIs of parameter values being statistically indistinguishable from zero (Table 5).

**Table 4:** WAIC results.

	*a_bone_*	*a_ind_*	*a_test_*	*b_cycle_*	WAIC	SE	dWAIC	dSE	pWAIC	Weight
m.04	X	X	X	O	869.9	26.62	0	NA	7.4	0.73
m.01	X	X	X	X	871.8	26.62	1.9	0.36	8.3	0.27
m.07	O	X	X	O	910.6	30.62	40.7	12.34	6.5	0
m.06	X	O	X	O	1,016	39.51	146.1	26.03	5	0
m.05	X	X	O	O	1030.7	38.75	160.8	21.37	6.8	0
m.03	X	O	O	O	1154.5	50.26	284.6	35.02	4.4	0
m.02	O	O	O	O	1184.1	53.64	314.2	38.85	3.5	0

X's and O's indicate inclusion and exclusion of the parameter from the model, respectively. For example, model m.06 included the effects of bone and test, but not individual or cycle. All models (labelled m.01–m.07) included an intercept (*a*) and the effect of device (*b_device_*). Models with lower WAIC values are more accurate. SE is the standard error in WAIC calculation, dWAIC and dSE are differences in WAIC scores and standard error between each model and the model with the highest weight, respectively, pWAIC is the effective number of parameters, and weight is the probability that is the best model of the models being compared.

**Table 5:** Average effect of the parameters in the models, 95% confidence intervals, and effective samples sizes for each variable.

	Median (95% CI)	Effective sample
Bone		
Femur	−0.04 (−1.31, 1.3)	948
Tibia	0.12 (−1.14, 1.46)	947
Individual		
1	0.27 (−0.22, 0.74)	2,236
2	−0.13 (−0.61, 0.34)	2,224
3	−0.09 (−0.57, 0.38)	2,238
4	−0.06 (−0.54, 0.41)	2,242
Test		
First	−0.22 (−1.86, 1.42)	619
Second	0.12 (−1.51, 1.76)	619
Intercept		
a	2.04 (−0.31, 4.41)	672
Slope (1 = Stryker, 2 = Imperial)		
b_device	−0.54 (−0.59, −0.49)	20,017

The 95% confidence intervals for bone, individual test (whether the device was tested first or second) and intercept of the model encompassed zero, indicating the parameters did not significantly affect these results at a significance level of α=0.05. For example, 0.27 indicates that Individual 1 increases interfragmentary motion by 0.27 mm, on average. Because the 95% CI is −0.22 to 0.74, and thus encompasses zero, the effects of Individual 1 on interfragmentary motion are not statistically significant.

## Discussion

The difference between the performance of the devices can be attributed to the difference in geometry and materials used in Imperial and Hoffmann 3 fixators. The Hoffman 3 is an excellent device, and our data suggests the Imperial fixator can provide similar levels of fracture fixation stiffness, using materials and manufacturing methods that are widely available in Low- and Middle-Income Countries.

The Bayesian mixed-effects model used here has enabled the effect of fixator type alone to be assessed accounting for testing sequence and specimens coming from one individual. Therefore, although this model predicts a median interfragmentary motion that is very similar to the median measurement taken from the raw data, the raw data 95% confidence intervals would have been inaccurate, as they would not have taken into account these confounding factors.

There have been many studies on low-cost external fixators, but most of them have reported the clinical outcomes (2, 26–28) often without performing thorough biomechanical investigations (10, 19, 29). This could be associated with the circumstances that the devices have been developed under, i.e., poor economical condition or conflict. Goh et al. (29) compared a low-cost fixator with a commercial AO device and reported that there was no significant difference between the stiffness of the devices. Similar to this research, Kouassi et al. (10) compared a different low-cost external fixator with Hoffmann 3 and reported that their device was significantly stiffer than Hoffmann 3.

Although there is a difference in device stiffness and there is the potential for this to change the local mechanical environment at the fracture site callus (30), this difference is small. A computational study found that such small differences have a negligible effect on bone healing (31).

Other factors are also important, including cost. The simple design and use of cheap and readily-available materials (aluminium and stainless steel) and conventional manufacturing processes result in the novel fixator (one rod and four clamps) costing a fraction of the cost of commercial devices. The device is 80 g heavier than Hoffmann 3. No advanced equipment or high level of manufacturing skills are needed when using the manufacturing toolkit. Due to the simplicity of the design and its similarity with Hoffmann 3, the device can simply be implemented with resident-level knowledge. Similar to Hoffmann 3, the novel device can also be used in uni- or bi-planar configurations.

This design and testing report has some limitations. Clinical results are not presented and these are required to confirm that the stiffness effects are not detrimental to healing. Also, the 95% CIs for the median interfragmentary motion values are large, reflecting the large level of noise in the data relative to the predictive parameters used in the statistical models. This could be because of the relatively low resolution of the interfragmentary motion data (±0.1mm) relative to the measurements taken. The design has been implemented primarily for lower limb long bone open fractures and so can accommodate one bone pin size (currently 5 mm for femur and tibia). Of course, if necessary, this could be easily adjusted during the manufacturing process to accommodate other pin sizes as might be required for upper limb applications. This report has also not yet presented results on its ability to be cleaned, sterilised and reused; this is the subject of an ongoing trial in Gaza on a gunshot wounds cohort. A second trial is underway in Sri Lanka and a third trial is underway in Ukraine (manufactured in Poland).

## Conclusion

There is clear evidence of a shortage of high-quality affordable external fixators in low- and middle-income countries, particularly in conflict and other unforeseen events. The affordable unilateral Imperial external fixator for long bone open fractures presented here has comparable performance to a commonly used commercial device and can be manufactured locally to international standards with minimal skills using basic materials and equipment when combined with the provided manufacturing toolkit. Three clinical studies are currently underway in Gaza, Sri Lanka and Ukraine.

## Data Availability

The original contributions presented in the study are included in the article/Supplementary Material, further inquiries can be directed to the corresponding author/s.
